# Supplementary material for: Stability and Bandgap Engineering of In1−xGaxSe Monolayer
Source: Nanomaterials (Basel). 2022 Feb 1;12(3):515. doi: 10.3390/nano12030515 (PMC8839788; doi:10.3390/nano12030515)
Supplement: Supplementary file 1 [file nanomaterials-12-00515-s001.zip › Supplementary/Supplementary.pdf]

Supporting Information for

Stability and Bandgap Engineering of $In_{1-x}Ga_xSe$ monolayer

Mattia Salomone[1], Federico Raffone[2], Michele Re Fiorentin[2], Francesca Risplendi[1] and Giancarlo Cicero[1]

[1] *Dipartimento di Scienza Applicata e Tecnologia, Politecnico di Torino, corso Duca degli Abruzzi 24, 10129 Torino, Italy*

[2] *Center for Sustainable Future Technologies, Istituto Italiano di Tecnologia, via Livorno 60, 10144 Torino, Italy*

1 Cluster Expansion results: Figures and ECIs

In this section we summarize all the results obtained through the Cluster Expansion (CE) method, performed by means of the Alloys Theoretic Automated Toolkit (ATAT) software [1]. In Figure S1 are reported all the *clusters* associated to pair interactions, while in table S1 are shown their related ECIs. For the Background (BG) and the Singlet (SG) interactions we reported only their energetic contribution.

**Type of interaction**	**ECI** (eV)
BG	-11206.995979
SG	-177.919832
NN1	-0.002100
NN2	0.068153
NN3	-0.004865
NN4	0.014843
NN5	-0.000915
NN6	0.029616
NN7	-0.000495
NN8	-0.009949
NN9	-0.000832
NN10	-0.012920
NN11	-0.000831
NN12	-0.029359
NN13	-0.000080
NN14	-0.024669
NN15	-0.000072
NN16	-0.024759

Table S1: Effective Cluster Interactions of the figures obtained from the CE method.

2 Zacros ECIs and lattice rearrangement

Kinetic Monte Carlo (KMC) simulations were performed exploiting the Zacros software [2,3] which is thought to deal with surface processes. In this work, we used this program to model the diffusion of gallium defects through the compound structure by means of positional exchange between Ga and In. The lattice is then composed by two stacked atomic layers (Se atoms are not taken into

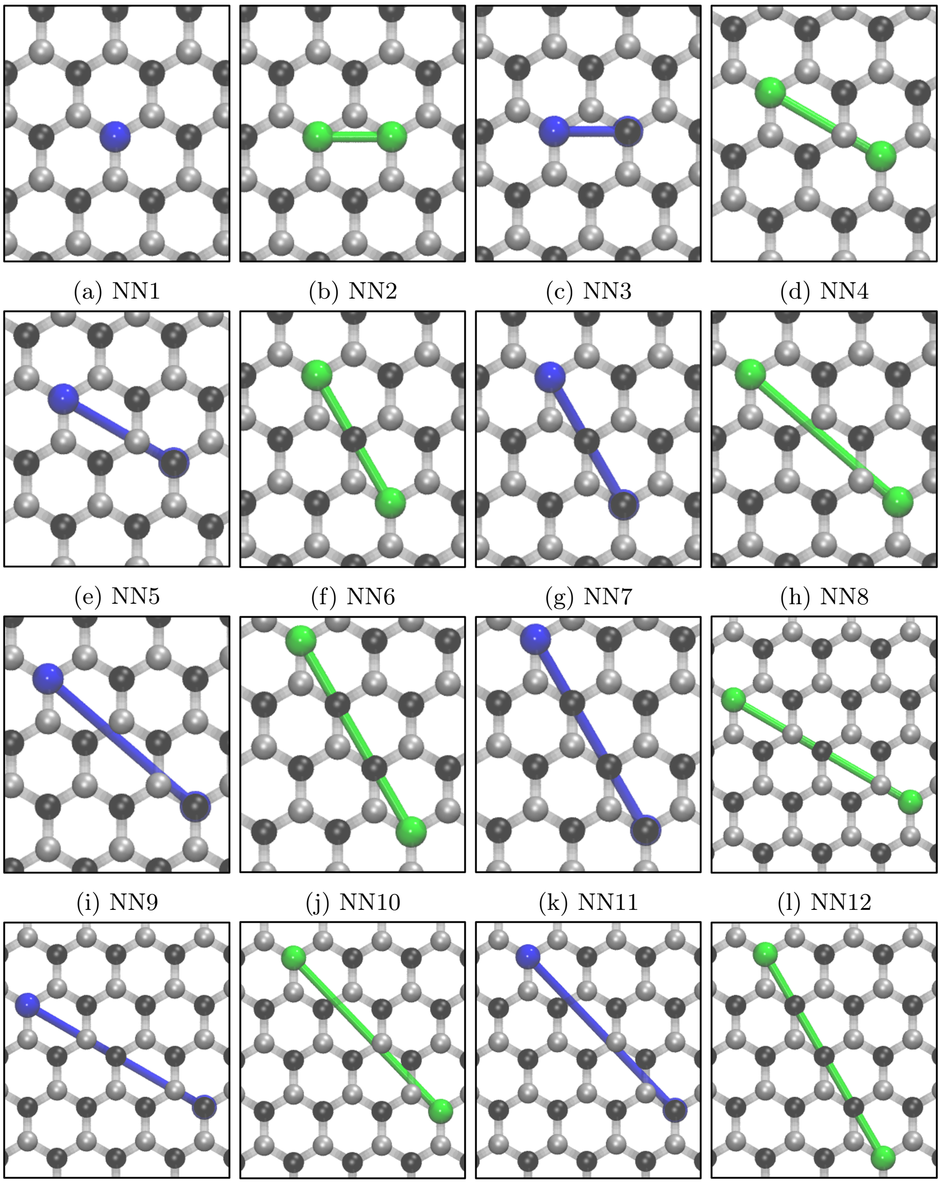

(a) NN1 (b) NN2 (c) NN3 (d) NN4
(e) NN5 (f) NN6 (g) NN7 (h) NN8
(i) NN9 (j) NN10 (k) NN11 (l) NN12
(m) NN13 (n) NN14 (o) NN15 (p) NN16

Figure S1: All the *pair interactions* obtained from the Cluster Expansion. Interplane (intraplane) figures are represented in blue (green).

account, because Ga substitutes only In).In Zacros we emulated the stacking of the two layers by defining a structure with a thickness of only one atom, made of unitary cells containing two sites placed at the same level (see Figure S2 as reference). To reproduce the 3D nature of the system, those sites were defined of two different types: *Top* (Figure S2, orange atoms) and *Bottom* (Figure S2, green atoms). We defined vertical transitions (i.e. from a site of type *Top* to a site of type *Bottom*) only between two sites belonging to the same unit cell (Figure S2, blue area) and horizontal exchanges only between nearest neighbours of the same types (*Top*/*Top* and *Bottom*/*Bottom*, green and orange bonds in Figure S2). A combination of these two processes (vertical transition between two different unit cells) was considered to be impossible. Zacros exploits results of the CE to calculate all the energetic parameters of the system during each simulation. However, its formalism is different with respect to the one adopted by ATAT. In the latter, indeed, each lattice site can be either in $+1$ or -1 state, while in Zacros $\sigma_{\mathrm{i}} = 0/1$ (where σ_{i} is the occupational variable of the i-th site). The conversion procedure can be found in reference In Figure S3 is highlighted

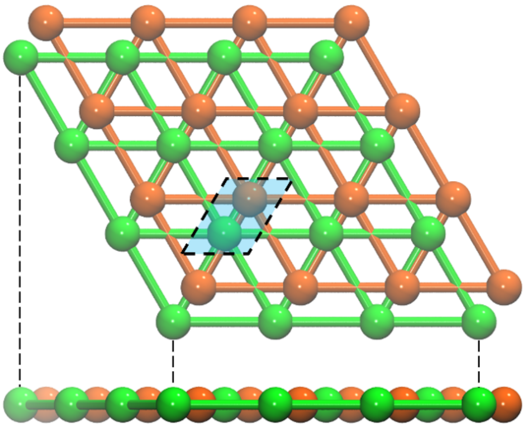

Figure S2: Atoms of type *Top* are represented in orange, while atoms of type *Bottom* in green. Transitions can occur only between nearest neighbours of the same type, except for atoms belonging to the same unitary cell (highlighted in blue).

the most frequent pattern that appear in the InGaSe lattice. It is almost totally determined by the repetition of pair figures NN12, NN14 and NN16 (green lines).

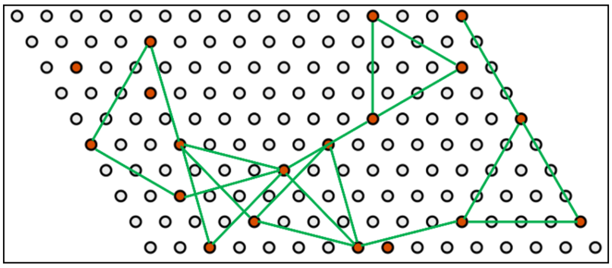

Figure S3: The three figures associated to the most negative ECIs are highlighted in green. It can be noticed that their repetition almost totally describes the gallium disposition within the lattice. For the sake of simplicity, only one of the two layers is represented.

3 Bowing parameter

The dependence of the energy gap on compound composition (shown in figure 5a) almost fits the quadratic form [4]:

$$\mathrm{E}_g(x) = (1-x)\mathrm{E}_g|_{\mathrm{InSe}} + x\mathrm{E}_g|_{\mathrm{GaSe}} - x(1-x)\mathrm{C}$$

where C is the so-called bowing parameter which accounts for the deviation from a linear interpolation between the pure phases of InSe and GaSe, and x is the Ga concentration. In our case C$= -0.23$ eV, calculated according to the literature [5], i.e. the compound bandgap is bigger than the linear interpolation result (Figure S4).

4 Optical properties

To investigate the possible appearance of in-plane anisotropies in InGaSe compounds, we have computed the optical absorption spectrum of some representative samples within the Independent Particle Random Phase Approximation (IP-RPA). In Figure S5 we report the imaginary part of the dielectric function of pristine InSe, $In_{0.83}Ga_{0.17}Se$, $In_{0.50}Ga_{0.50}Se$, $In_{0.17}Ga_{0.83}Se$ and pristine GaSe. The red and green curves respectively mark the absorption of light polarized along the the zig-zag (x) and armchair (y) directions of the hexagonal lattice. It is clear from our results that InGaSe compounds do not show in-plane optical anisotropy.

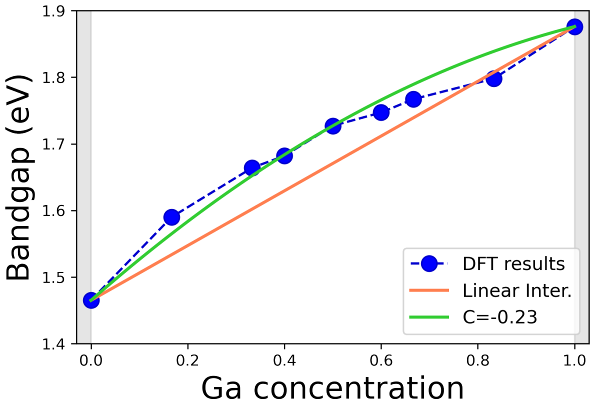

Figure S4: The red line indicates the linear interpolation of the DFT-obtained energy gap behaviour, while the green curve is obtained when the bowing parameter C is considered.

References

[1] A. van de Walle, G. Ceder, *J Phase Equilib* **23**, 348 (2002).

[2] J. Nielsen, M. D'Avezac, J. Hetherington, M. Stamatakis, *J Chem Phys* **224706**, 139 (2013).

[3] M. Stamatakis, D. G. Vlachos, *J Chem Phys* **134**, 214115 (2011).

[4] I. Vurgaftman, J. R. Meyer, L. R. Ram-Mohan, *J Appl Phys* **89**, 5815 (2001).

[5] J. A. V. Vechten, T. K. Bergstresser, *Phys Rev B* **1**, 3351 (1970).

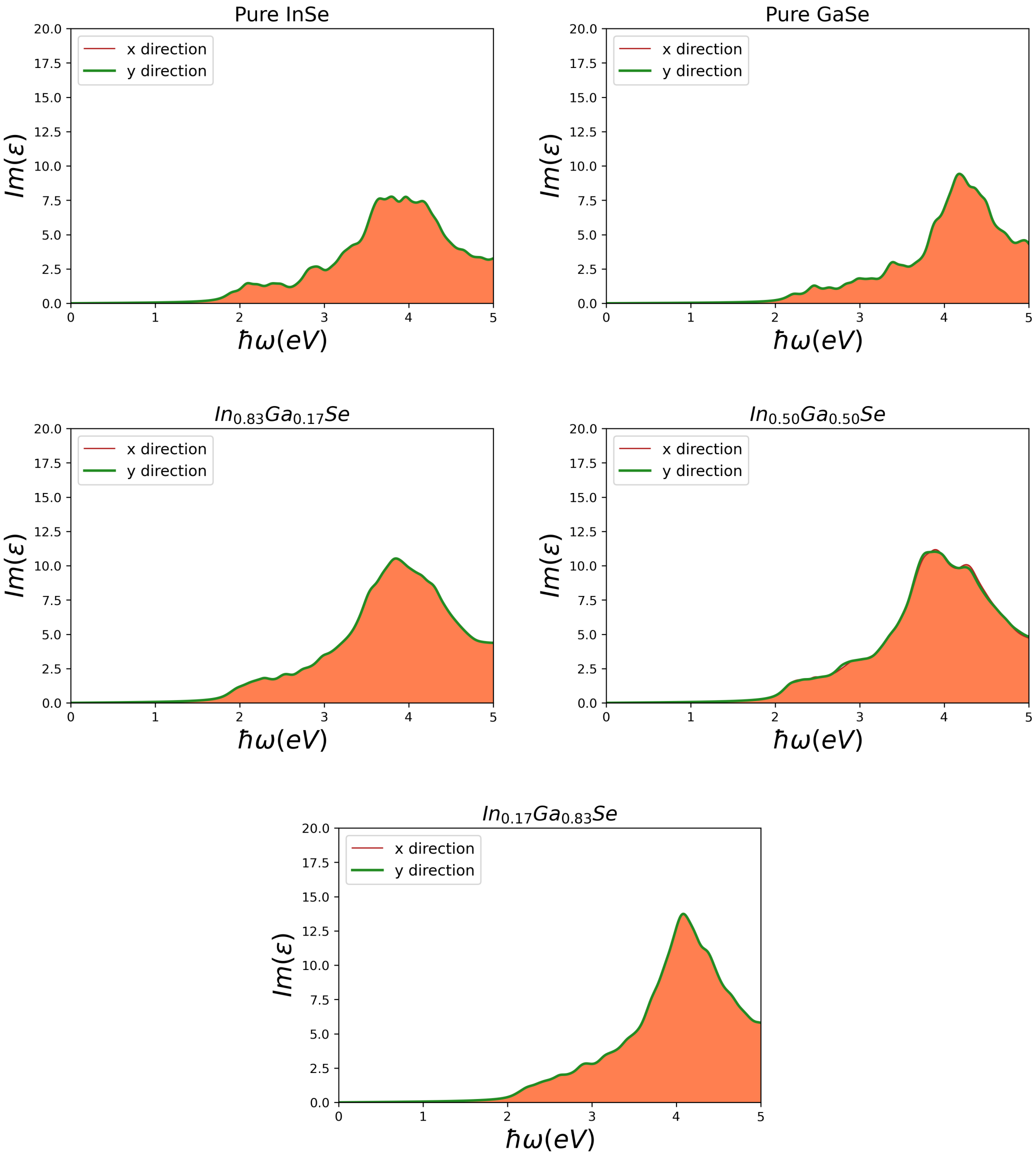

Figure S5: The red and green curves represent Im(ε) along the x and y directions respectively. The two curves clearly overlap, thus optical absorption appears to be an isotropic quantity.
