# Supplementary figures and images for: Stability and Bandgap Engineering of In1−xGaxSe Monolayer
Source: Nanomaterials (Basel). 2022 Feb 1;12(3):515. doi: 10.3390/nano12030515 (PMC8839788; doi:10.3390/nano12030515)

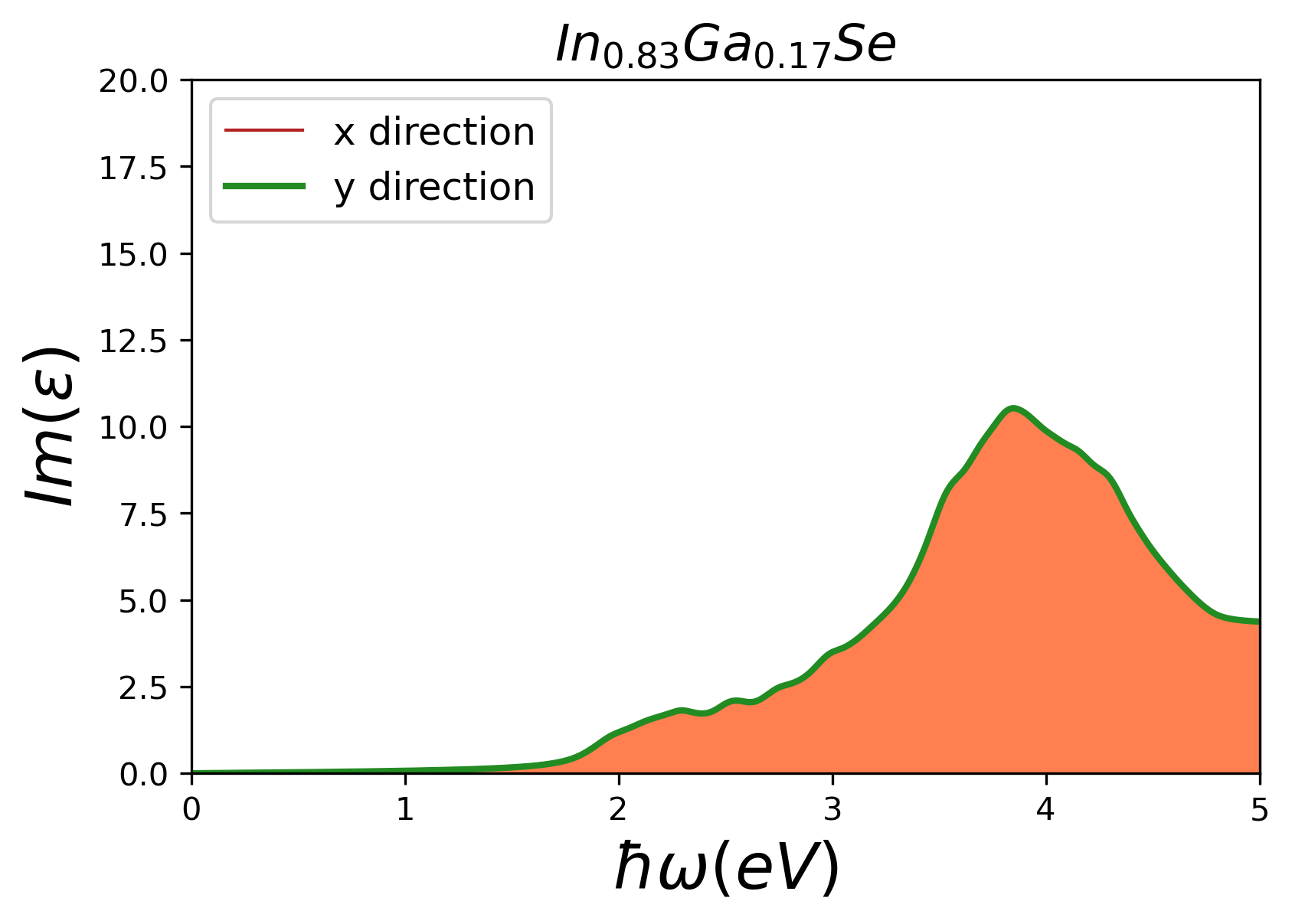

Supplement: Supplementary file 1 [file nanomaterials-12-00515-s001.zip › Supplementary/17x100_epsi.png]

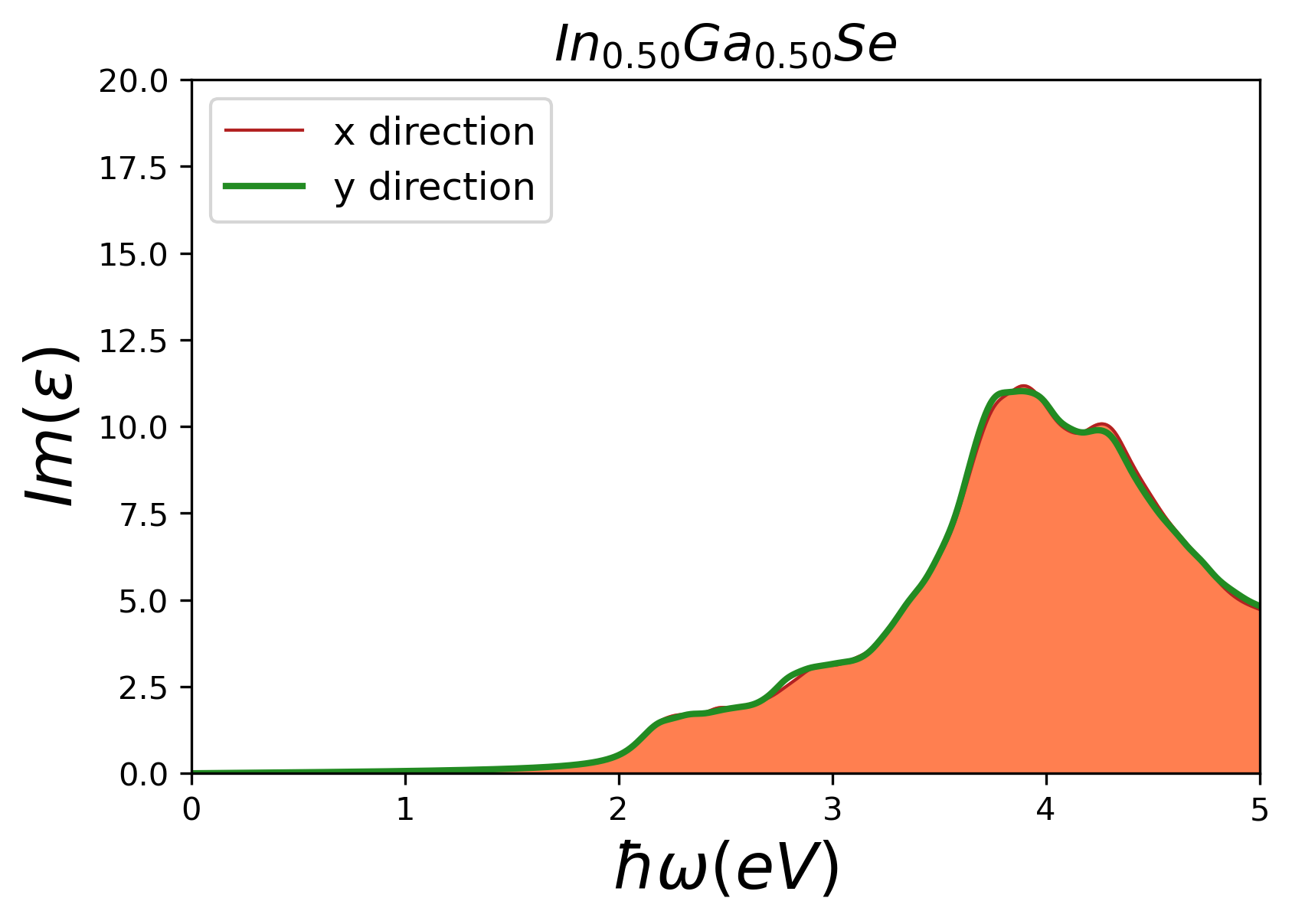

Supplement: Supplementary file 1 [file nanomaterials-12-00515-s001.zip › Supplementary/50x100_epsi.png]

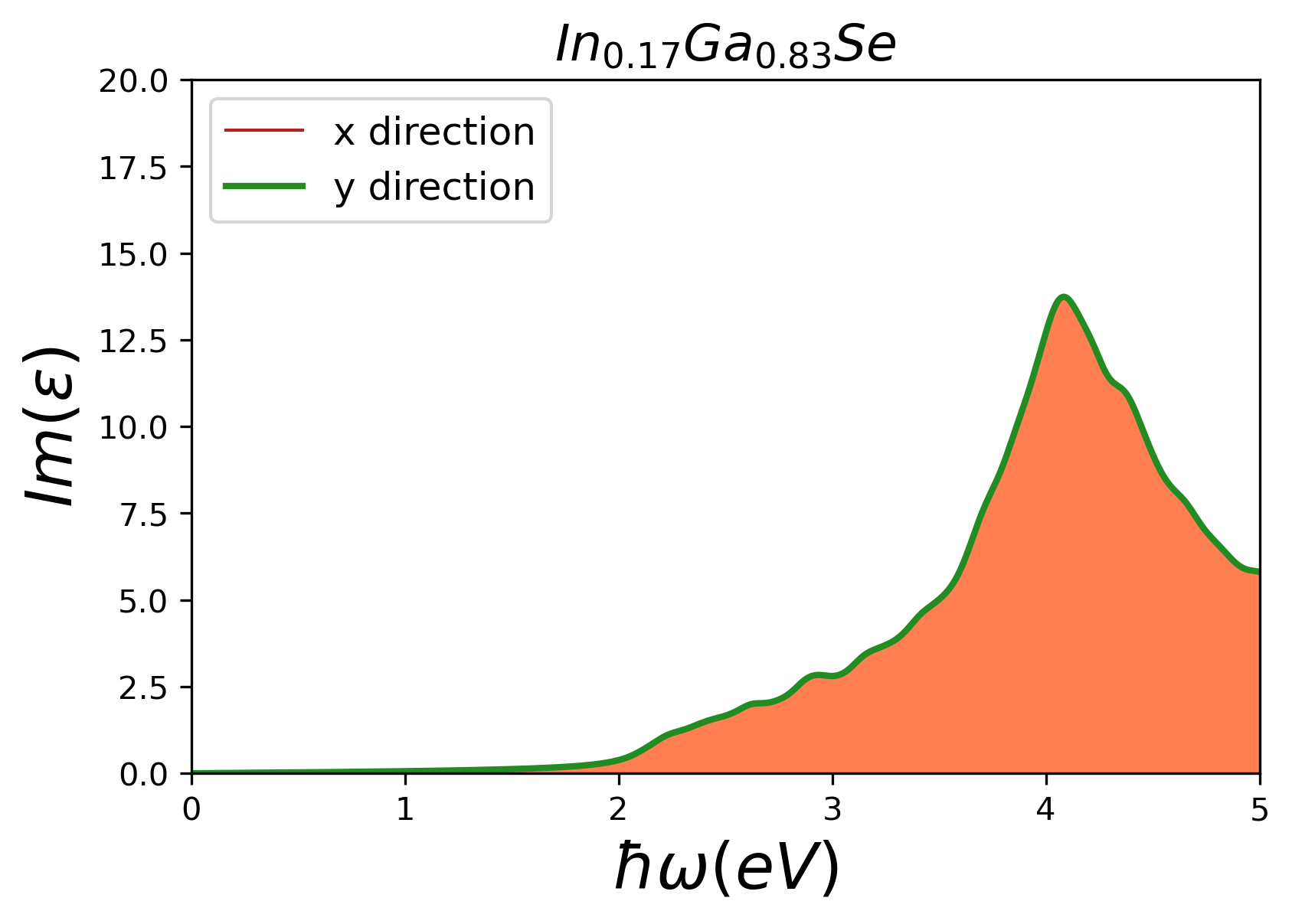

Supplement: Supplementary file 1 [file nanomaterials-12-00515-s001.zip › Supplementary/83x100_epsi.png]

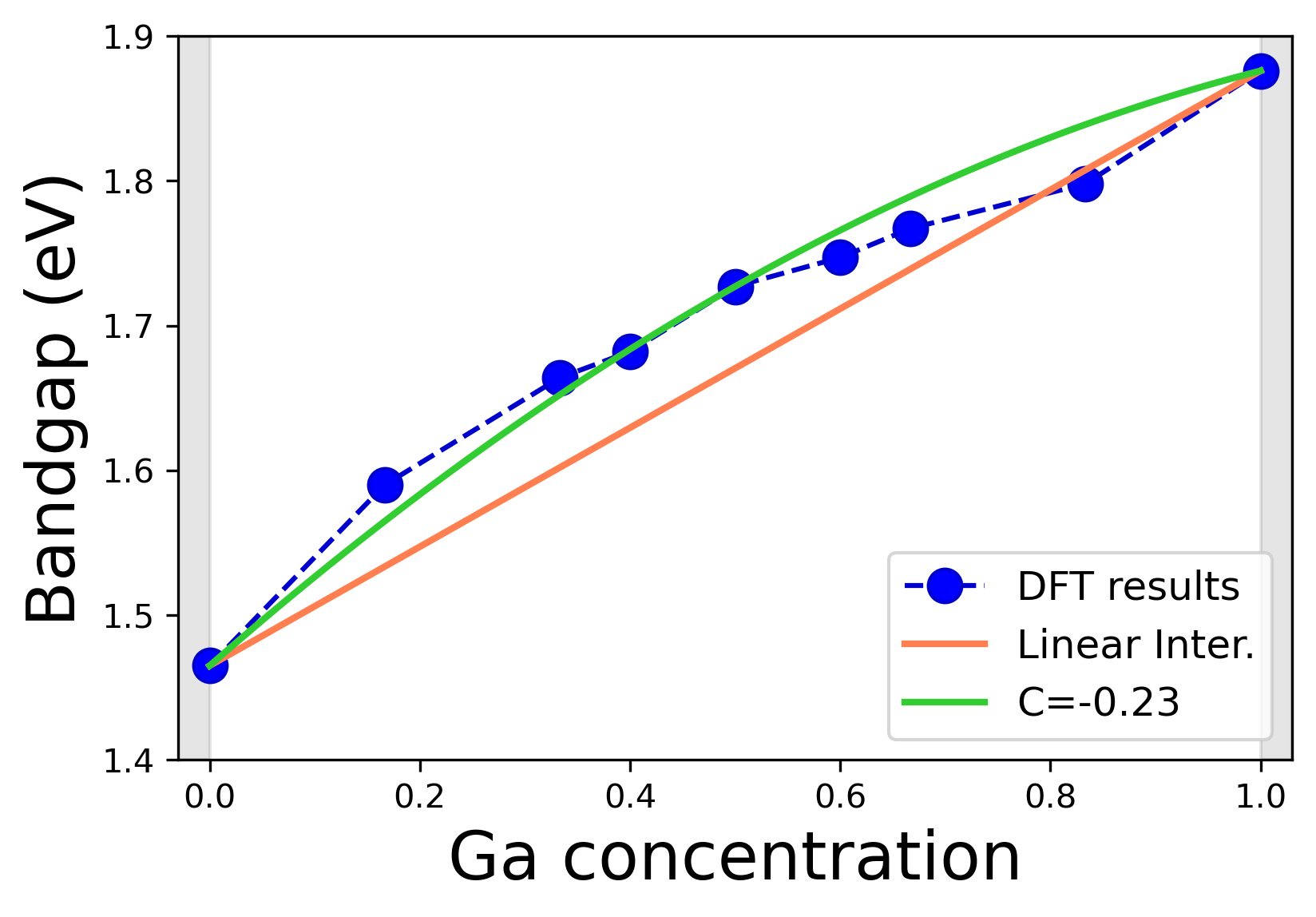

Supplement: Supplementary file 1 [file nanomaterials-12-00515-s001.zip › Supplementary/Bowing_Parameter.png]

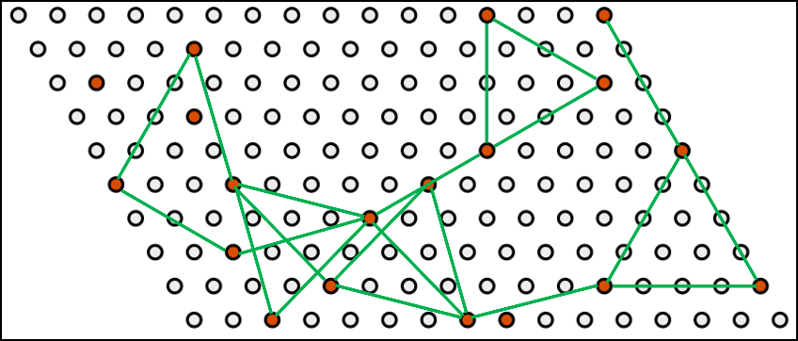

Supplement: Supplementary file 1 [file nanomaterials-12-00515-s001.zip › Supplementary/Figure_SHOWN.png]

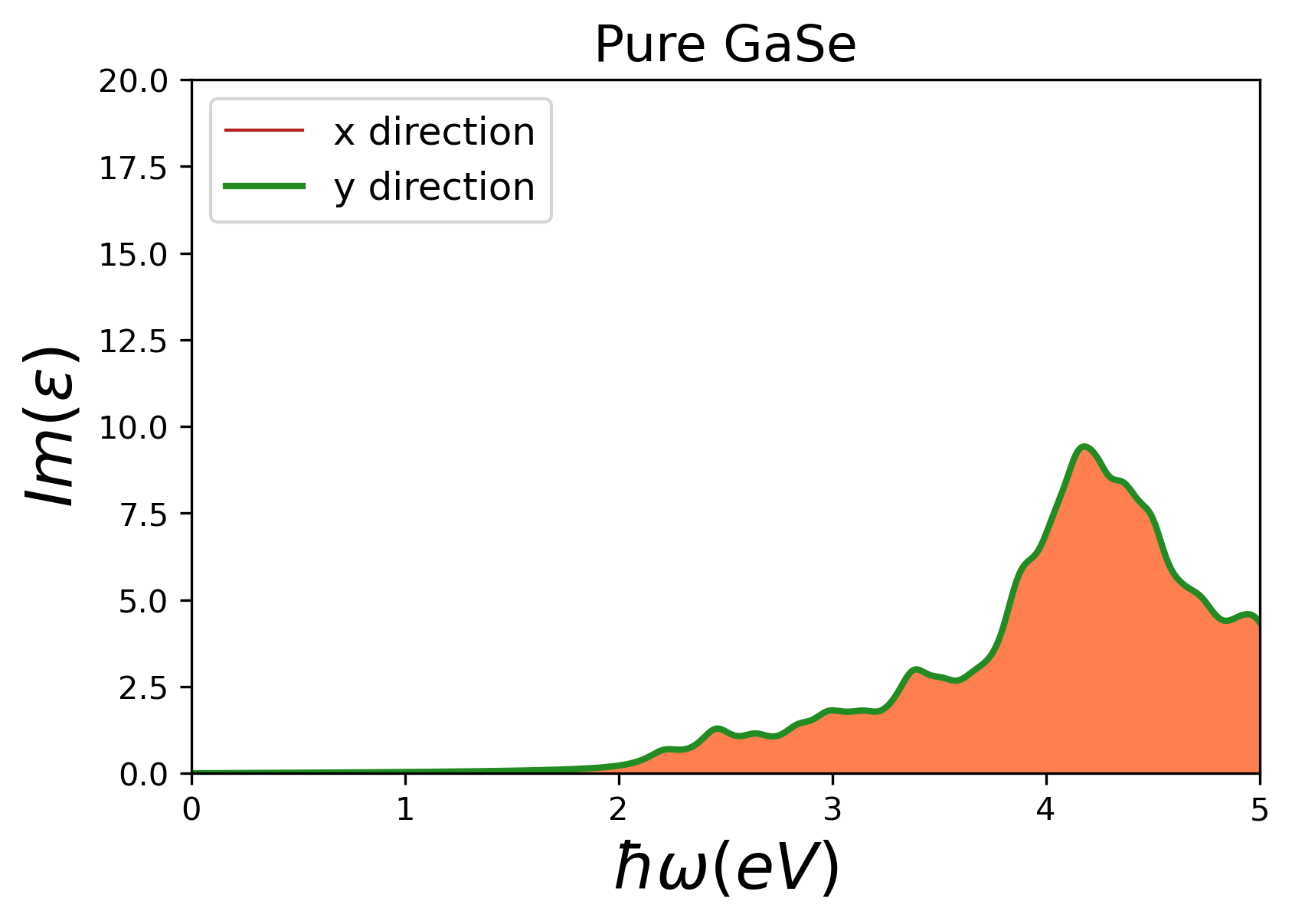

Supplement: Supplementary file 1 [file nanomaterials-12-00515-s001.zip › Supplementary/GaSe_epsi.png]

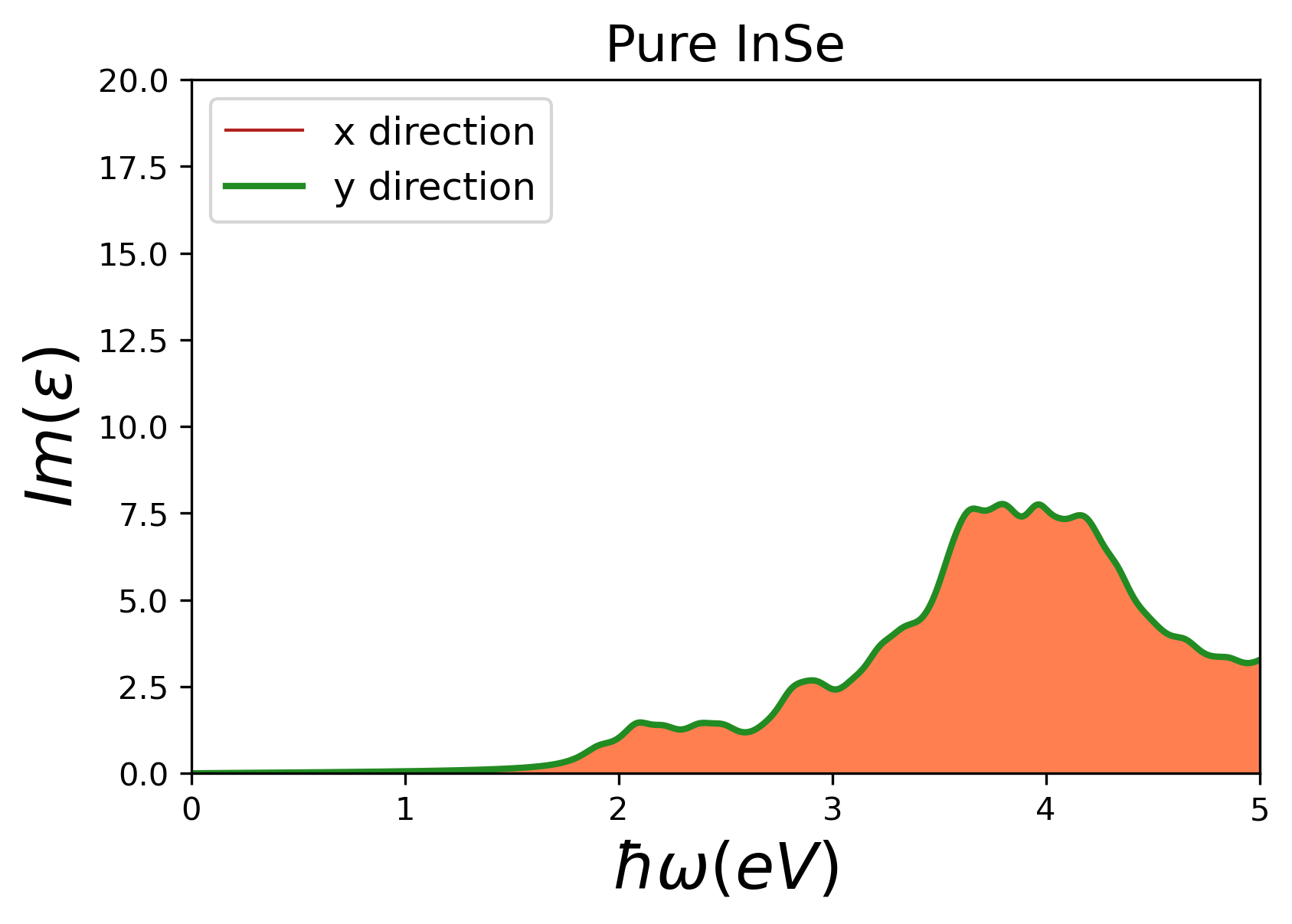

Supplement: Supplementary file 1 [file nanomaterials-12-00515-s001.zip › Supplementary/InSe_epsi.png]

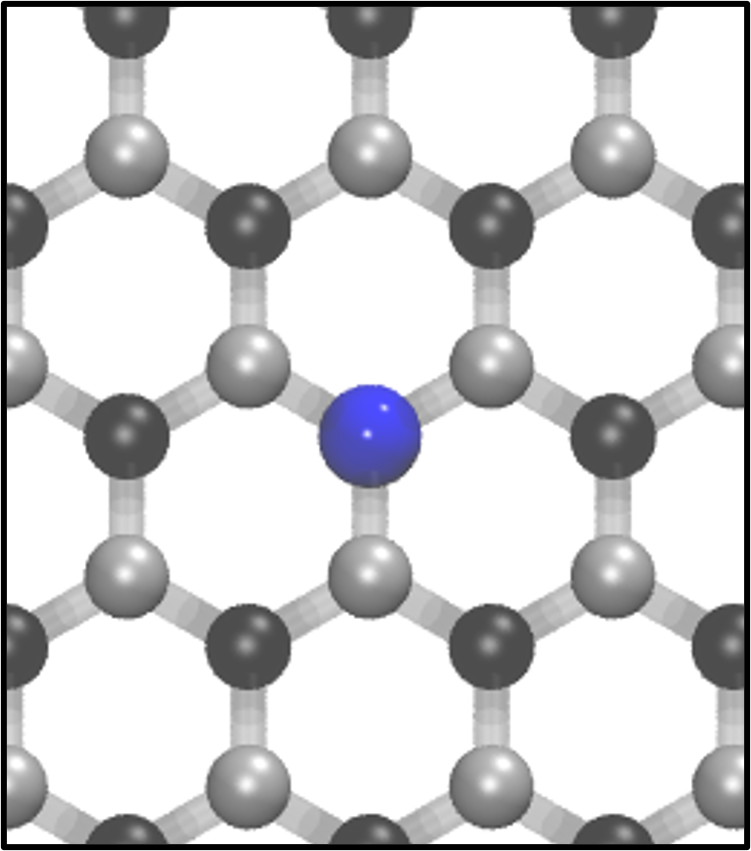

Supplement: Supplementary file 1 [file nanomaterials-12-00515-s001.zip › Supplementary/Pair1.png]

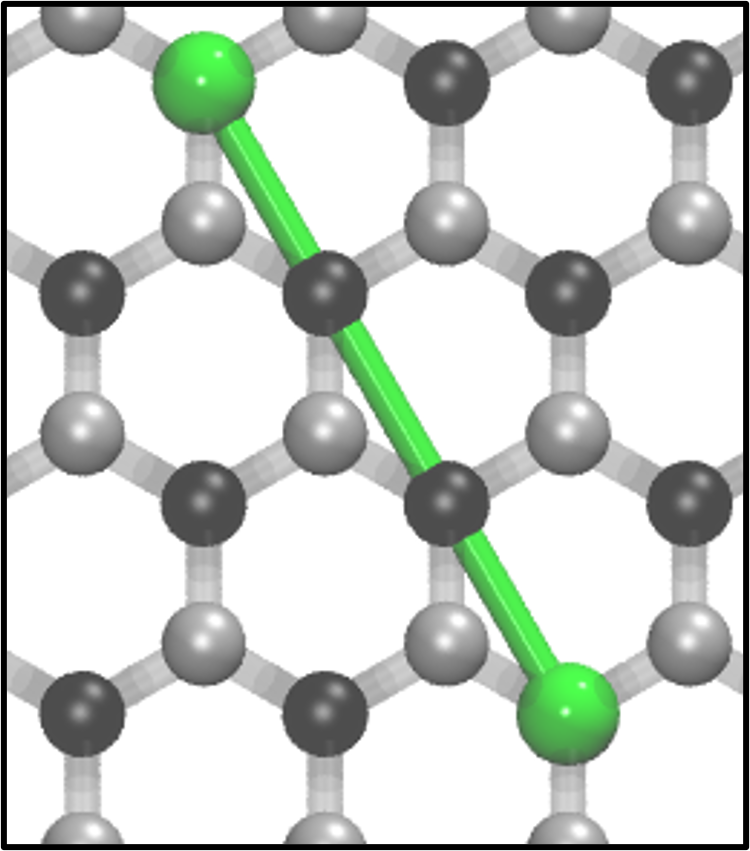

Supplement: Supplementary file 1 [file nanomaterials-12-00515-s001.zip › Supplementary/Pair10.png]

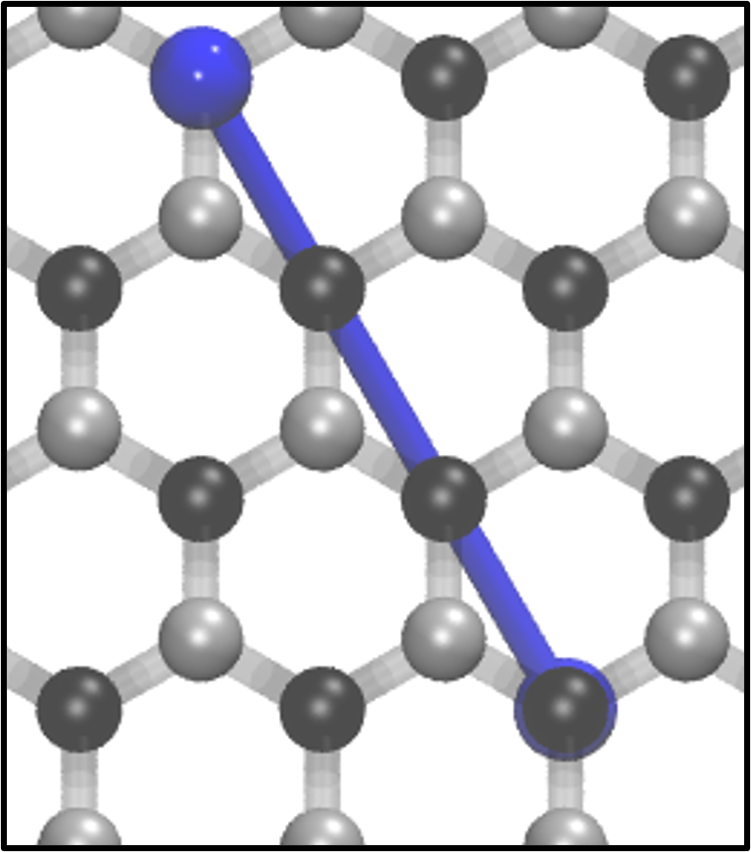

Supplement: Supplementary file 1 [file nanomaterials-12-00515-s001.zip › Supplementary/Pair11.png]

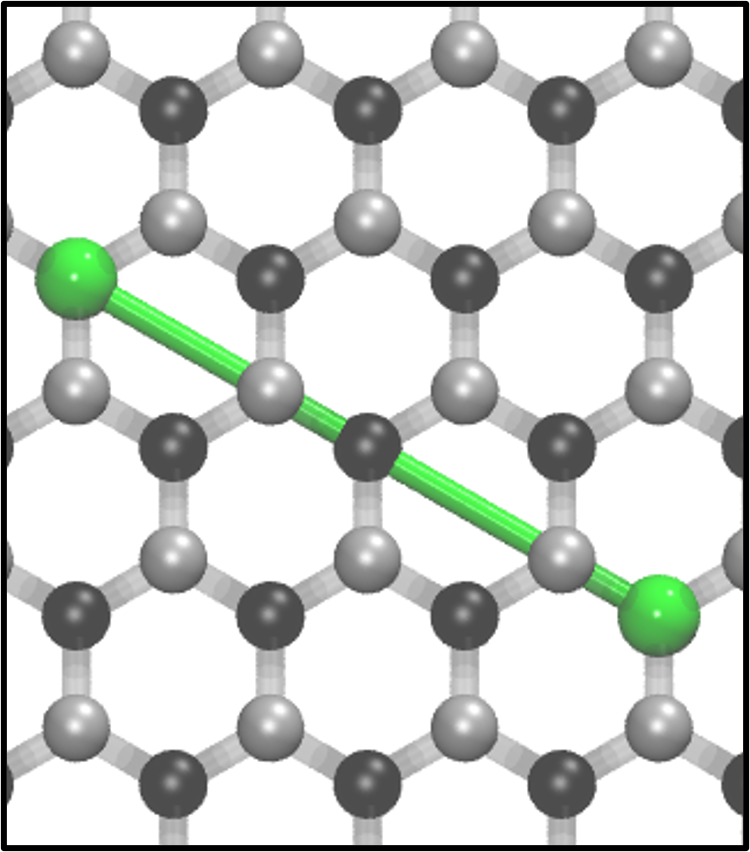

Supplement: Supplementary file 1 [file nanomaterials-12-00515-s001.zip › Supplementary/Pair12.png]

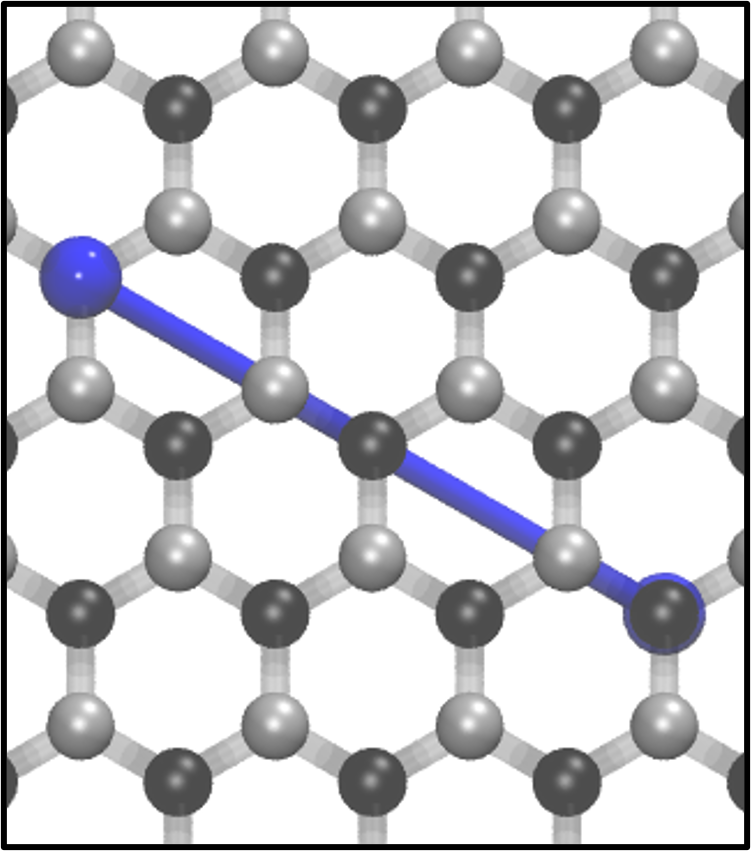

Supplement: Supplementary file 1 [file nanomaterials-12-00515-s001.zip › Supplementary/Pair13.png]

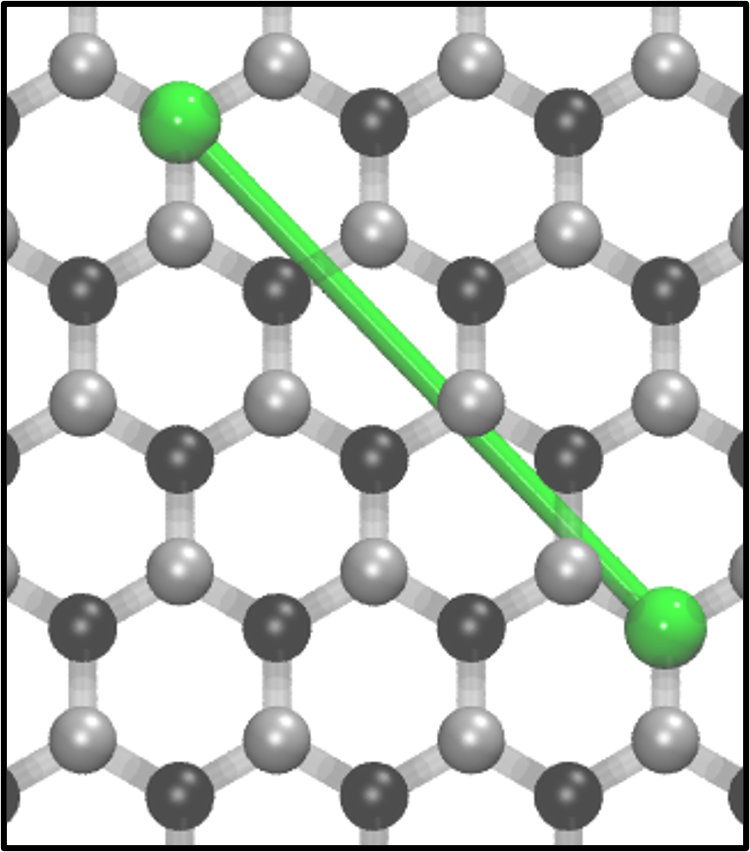

Supplement: Supplementary file 1 [file nanomaterials-12-00515-s001.zip › Supplementary/Pair14.png]

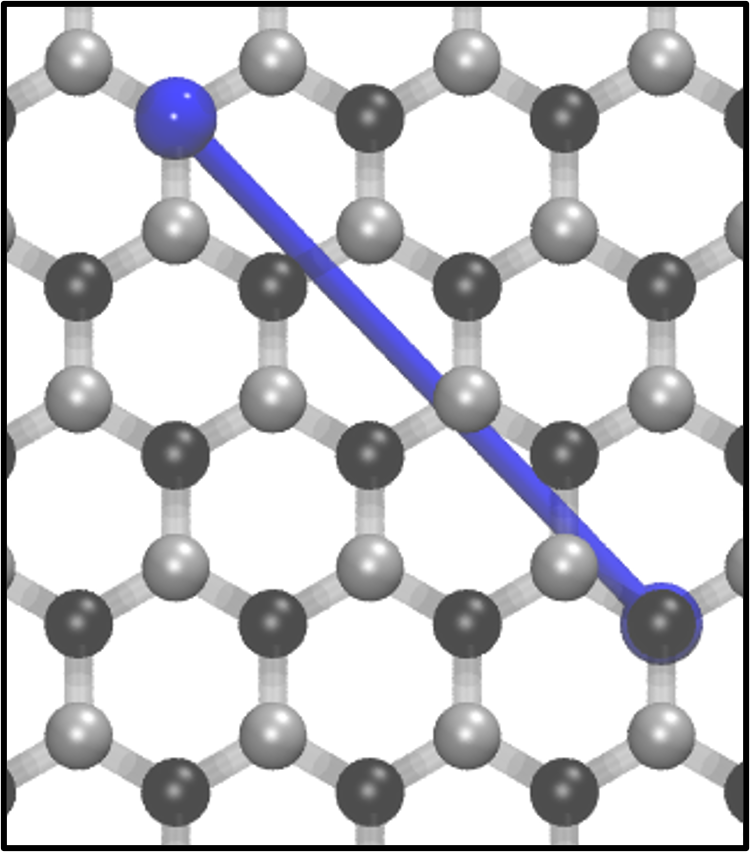

Supplement: Supplementary file 1 [file nanomaterials-12-00515-s001.zip › Supplementary/Pair15.png]

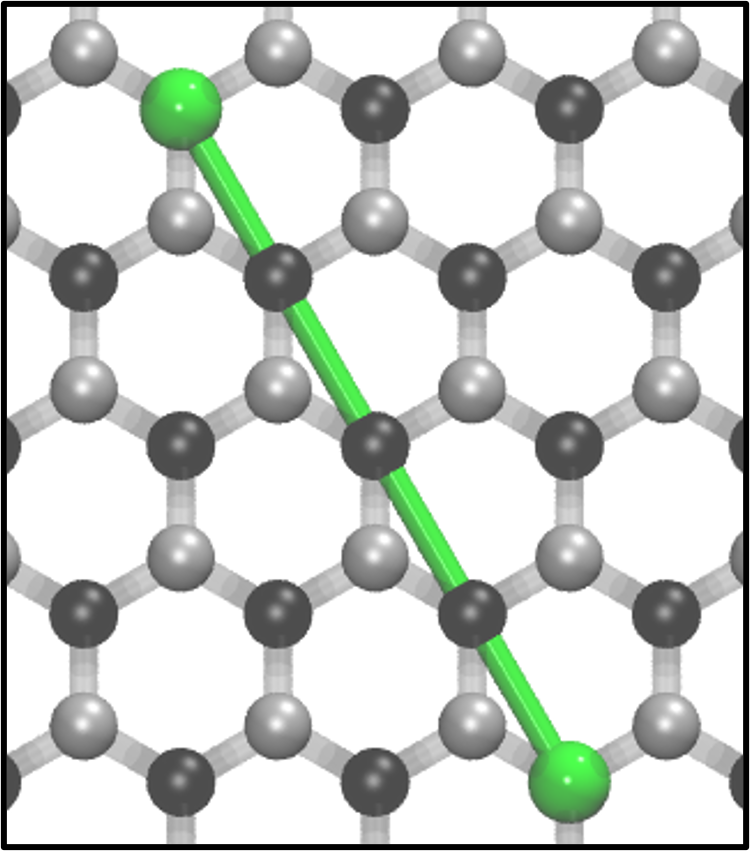

Supplement: Supplementary file 1 [file nanomaterials-12-00515-s001.zip › Supplementary/Pair16.png]

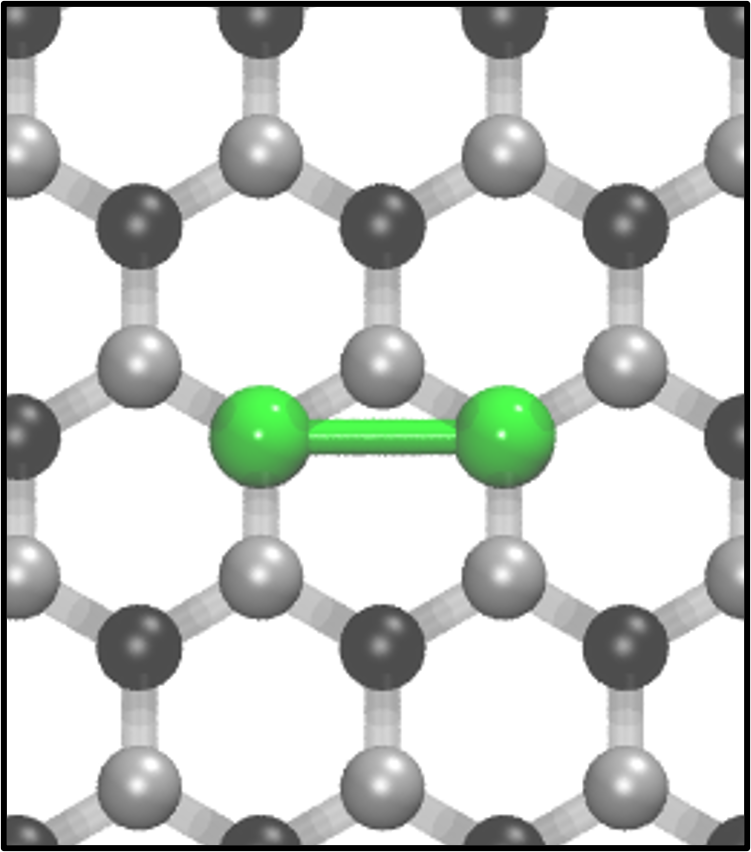

Supplement: Supplementary file 1 [file nanomaterials-12-00515-s001.zip › Supplementary/Pair2.png]

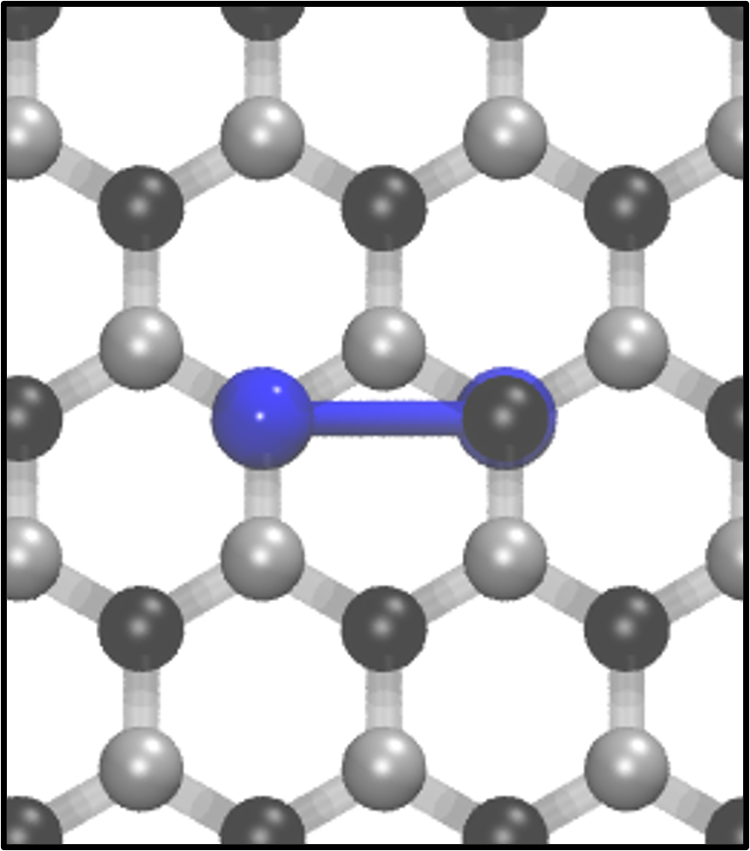

Supplement: Supplementary file 1 [file nanomaterials-12-00515-s001.zip › Supplementary/Pair3.png]

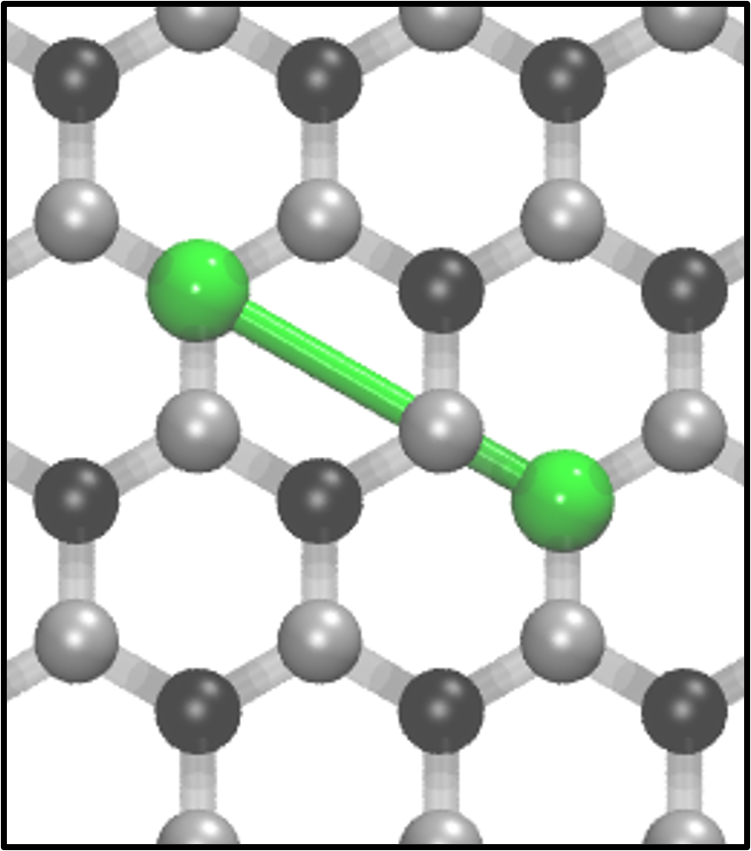

Supplement: Supplementary file 1 [file nanomaterials-12-00515-s001.zip › Supplementary/Pair4.png]

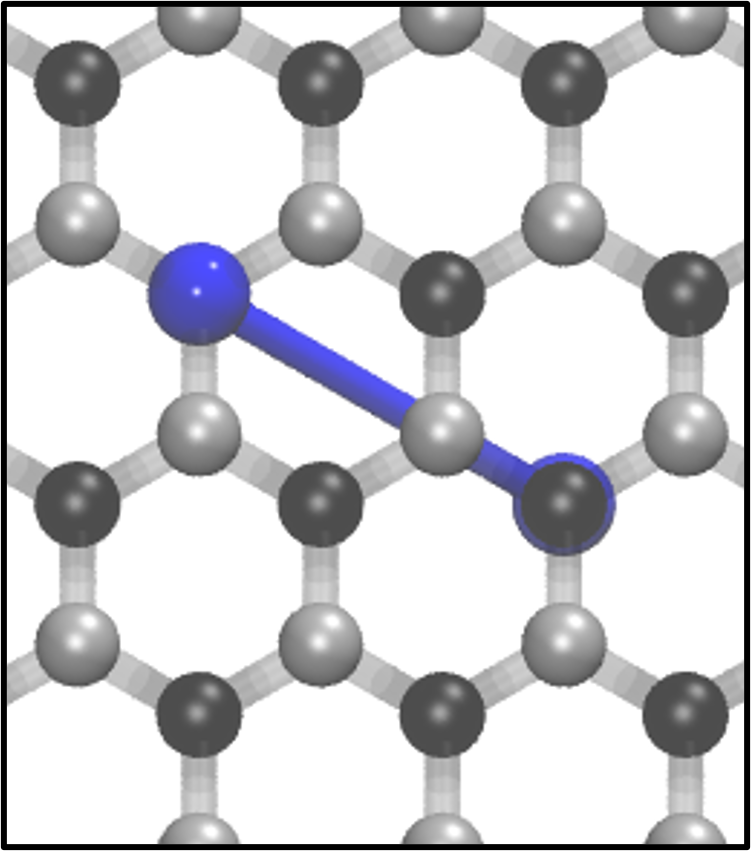

Supplement: Supplementary file 1 [file nanomaterials-12-00515-s001.zip › Supplementary/Pair5.png]

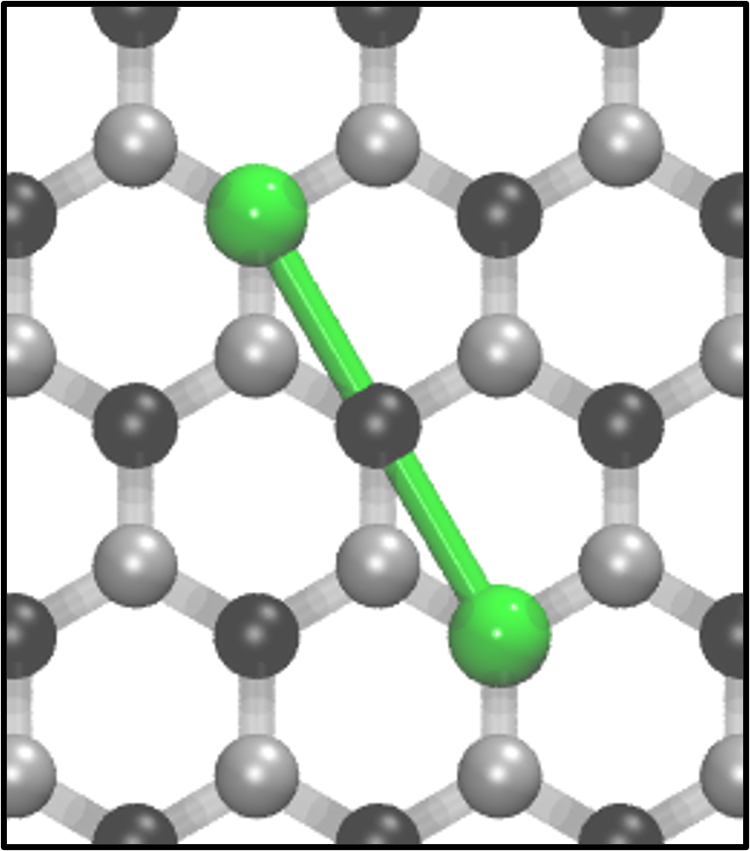

Supplement: Supplementary file 1 [file nanomaterials-12-00515-s001.zip › Supplementary/Pair6.png]

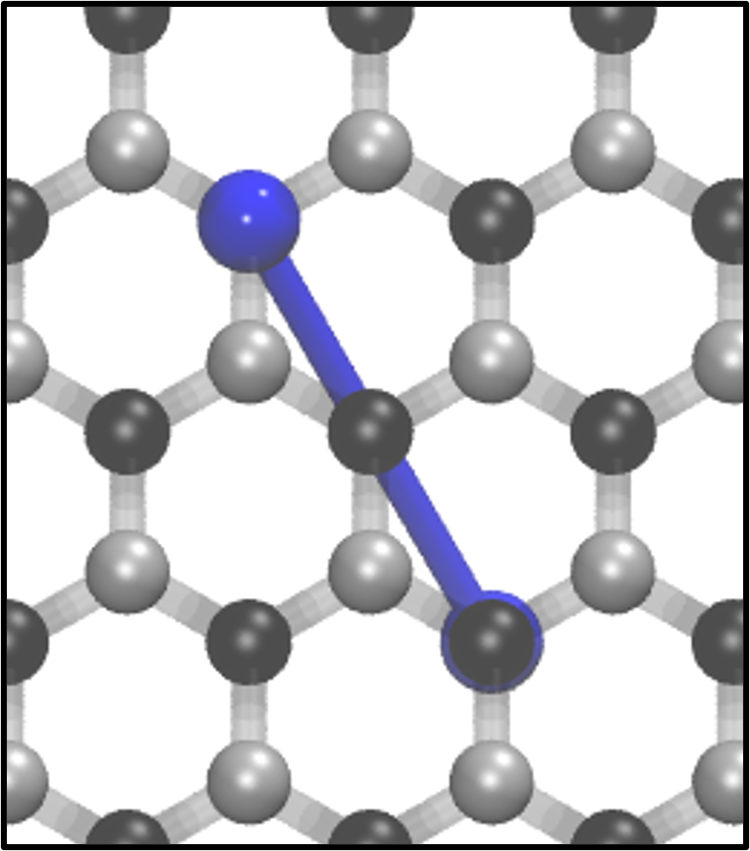

Supplement: Supplementary file 1 [file nanomaterials-12-00515-s001.zip › Supplementary/Pair7.png]

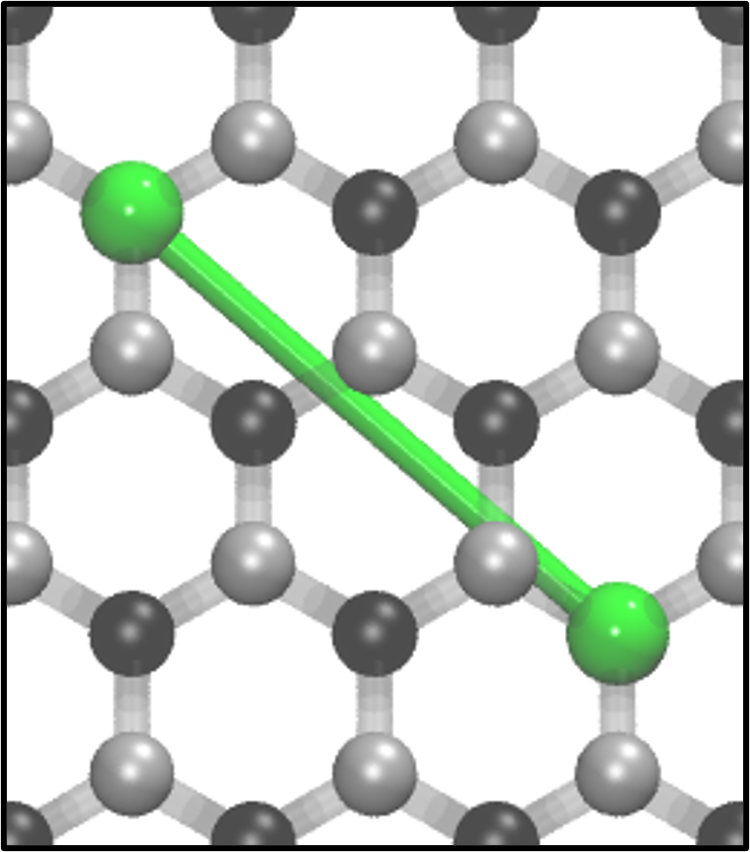

Supplement: Supplementary file 1 [file nanomaterials-12-00515-s001.zip › Supplementary/Pair8.png]

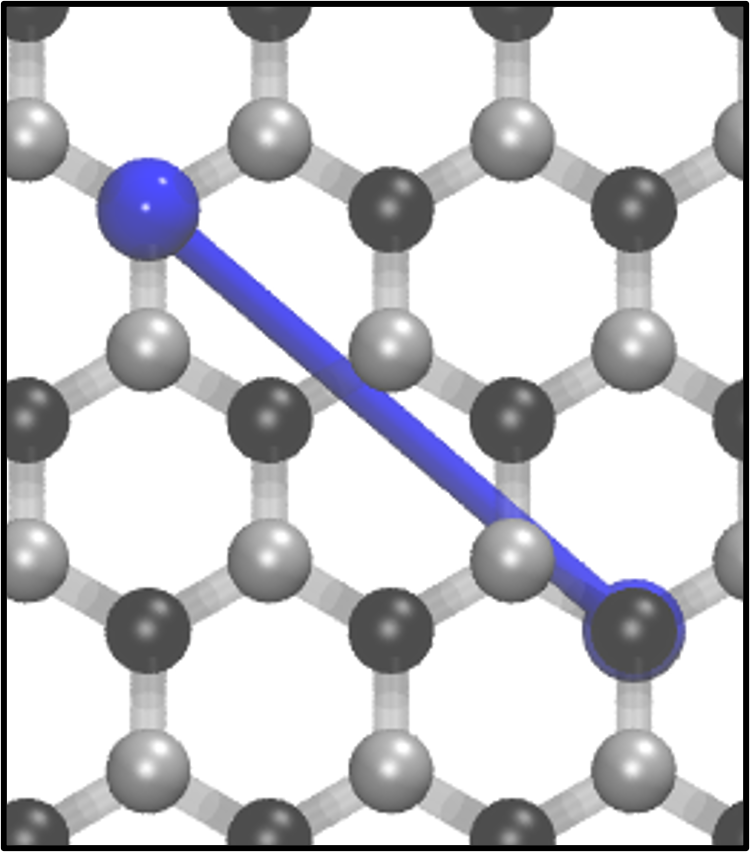

Supplement: Supplementary file 1 [file nanomaterials-12-00515-s001.zip › Supplementary/Pair9.png]

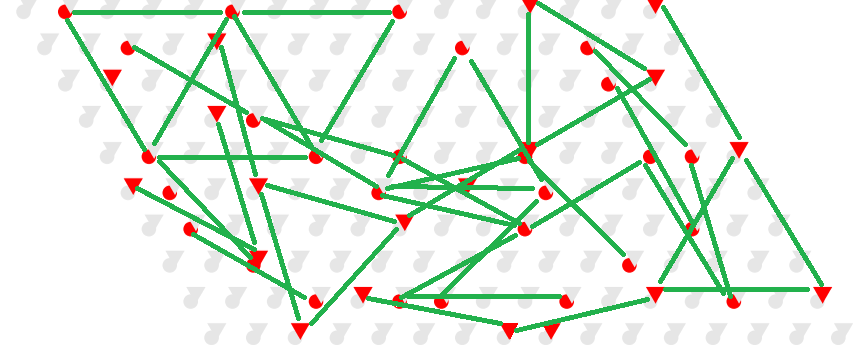

Supplement: Supplementary file 1 [file nanomaterials-12-00515-s001.zip › Supplementary/Provvisorio1_15.png]

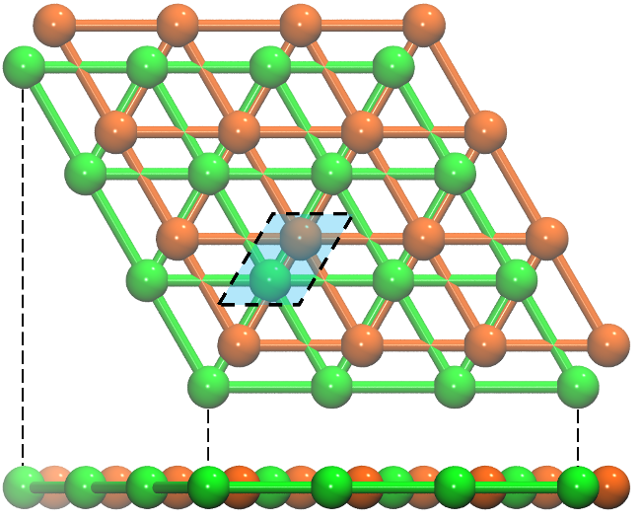

Supplement: Supplementary file 1 [file nanomaterials-12-00515-s001.zip › Supplementary/Transitions.png]
